# Supplementary material for: Comparison of delivery outcomes in low-dose and high-dose oxytocin regimens for induction of labor following cervical ripening with a balloon catheter: A retrospective observational cohort study
Source: PLoS One. 2022 Apr 22;17(4):e0267400. doi: 10.1371/journal.pone.0267400 (PMC9032418; doi:10.1371/journal.pone.0267400)
Supplement: Attachment — Submitted filename: Response to Reviewers.docx [file pone.0267400.s001.docx]

February 12^th^, 2022

Dear Reviewers,

Thank you for your comments on our manuscript titled "Comparison of delivery outcomes in low-dose and high-dose oxytocin regimens for induction of labor following cervical ripening with a balloon catheter" (PONE-D-21-36119, EMID e1b9956a3ea09791). We are pleased to learn the manuscript may be after a revision considered for publication. We have carefully considered the comments and concrns and revised the manuscript accordingly. We appreciate the time and effort that you as reviewers have dedicated to providing your valuable feedback on the manuscript. Please find the point-by-point response to the comments below. The changes in the manuscript have been indicated using tracked changes. The line numbers mentioned in this letter refer to the clean copy of the manuscript.

**Journal requirements and the authors’s response:**

1. Please ensure that your manuscript meets PLOS ONE's style requirements, including those for file naming. The PLOS ONE style templates can be found at

<https://journals.plos.org/plosone/s/file?id=wjVg/PLOSOne_formatting_sample_main_body.pdf>  and

<https://journals.plos.org/plosone/s/file?id=ba62/PLOSOne_formatting_sample_title_authors_affiliations.pdf>

**Authors’s response:** We have now carefully studied the journal’s style requirements and formatted the manuscript and file names accordingly.

2. Please provide additional details regarding participant consent. If you are reporting a retrospective study of medical records, archived samples or third party data, please ensure that you have discussed whether all data were fully anonymized before you accessed them. Once you have amended this/these statement(s) in the Methods section of the manuscript, please add the same text to the “Ethics Statement” field of the submission form.

**Authors’s response:** The additional details have been added in the Methods and Ethics statement

**Reviewer’s comments to the authors and authors’ response**

**Reviewer #1
Major issues:**

**1. Title:** It would be recommended for authors to add the study design in the title. For example: “Comparison of delivery outcomes in low-dose and high-dose oxytocin regimens for induction of labor following cervical ripening with a balloon catheter: A retrospective observational cohort study.”

**Authors’s response:** Thank you, this has been done.

**2. Abstract:**
a. Methods: Specify how many patients in each group i.e. low-dose oxytocin protocol (n= x) and high dose oxytocin protocol (n= y).

**Authors’s response**: Number of patients in each protocol have now been added in the methods section of the Abstract (lines 6-7).

b. Results: Authors should add induction to delivery interval in results section of abstract as results from other secondary outcomes are stated.

**Authors’s response**: The median (IQR) induction-to-delivery interval results have been added in the results section of the Abstract (lines 21-22). To still meet the limited 300-word count of the Abstract after these additions, we have slightly edited the abstract. All the removed words have been indicated with tracked changes.

**3. Tables and figures:**
a. Table 3: There are 206 patients in new protocol group in Table 3 as well as in Figure ,1 whereas the text states it is 207 patients. Adding a flow chart in the results section may be useful in clarifying this.

**Authors’s response:** There has been an unfortunate typo. The correct number of patients in the new protocol is 207, and this has now been corrected in Table 3 and Figure 2 (former Fig 1). We have also added a flow chart in the Results section (the new Fig 1).

b. Table 4: Table 3 has total number of patients in each group at top of the table but table 4 does not.

**Authors’s response:** The total number of patients has been added in each group at top of Table 4.

c. Table 4: Authors should clarify the maternal infection value in old protocol (n=62) and subgroups intrapartum infection (n=46) and postpartum infection (n=18) as they do not add up. Furthermore, it might be useful to mention that the subgroups of maternal infection (intrapartum and postpartum infection) are not significantly different between the two protocols.

**Authors’s response:** Maternal intrapartum infection occurred in 62 women and post-partum infection in 46 women in the old protocol. Two of the women who had an intrapartum infection presented with a separate post-partum cesarean wound infection later, a few days after being discharged from the hospital. Both women had antibiotic treatment and revision surgery. This has now been clarified in the footnotes of Table 4.

d. Table 4: It would be useful to also present CTG changes/fetal distress regardless of mode of delivery if data is available.

**Authors’s response:** The authors regret that we don’t have the data on hyperstimulation as discussed in the limitations of the study. CTG changes and fetal distress leading to emergency cesarean delivery have been recorded, however, unfortunately the data on CTG changes in women who had vaginal delivery were not recorded. We have now emphasized this in the limitations paragraph of the Discussion (lines 282-286).

**4. Methods:**
a. If the authors have used the STROBE guidelines, please mention it in the methods. If it has not been used, please report this study in compliance with STROBE statement.

**Authors’s response**: The STROBE guideline has been used, and this is now stated in the Methods (lines 81-82).

b. It would be recommended to state if any efforts were made to address potential risk of bias.
**Authors’s response:** The authors acknowledge the potential bias being the retrospective design and the decision to include only women undergoing induction of labor at term, rather than at any time during the third trimester. We tried to address this bias by choosing two identical cohorts within two identical time periods during the two different oxytocin protocols. This has been discussed in the limitation of the study (lines 278-282), we now added it in the Methods as well (lines 71-74).

**5. Results:**
a. Could authors report numbers of individuals at each stage of study (examined for eligibility, confirmed eligible etc).

**Authors’s response:** We identified 3290 and 3085 deliveries during the selected time periods of the old and new protocol, examined 743 and 853 women for eligibility, and confirmed 280 and 207 women eligible, respectively. This has now been added in the beginning of the Results (157-159). We have also added the flowchart (Fig 1).

b. Page 14 lines 197-199: “In the old protocol, maternal or neonatal infection occurred in 29.3 % (n=82) of the labors, while in the new protocol the corresponding rate was 14.6 % (n=21); p<0.001”. This does not match results presented in table 4. In table 4 maternal or neonatal infection in the old protocol is reported in 82 patients (29.3%), and in 30 patients (14.6%) in the new protocol with a p value <0.0001.

**Authors’s response:** Thank you for pointing this out, the mistake (n=21 instead of n=30) and p> 0.001 in the the Results and Table 4 have been corrected (line 190).

c. Page 14 lines 201-203: The authors report that “The median birthweight, the rates of post-partum hemorrhage and umbilical artery blood pH-value <7.05 did not differ between the cohorts (Table 4).” However, in table 4 the mean SD birthweight is shown.

**Authors’s response:** Thank you, this sentence has now been corrected, the birthweight is expressed as mean (SD), not median (line 192).

**6. Discussion:**
a. Page 17 lines 236-239 “The rate of vaginal delivery was higher, and the rates of maternal and neonatal infections were lower during the new protocol. After the implementation of the new protocol, the rate of cesarean delivery in induced labor has decreased while the rate of labor induction is increasing.” It would be useful for the authors to add p values.

**Authors’s response**: We have now added the p-values and the rates to this sentence in the Discussion (lines 229-231).

**7. Conclusion:**
a. Page 20 lines 295-296: “No difference in complications, such as post-partum hemorrhage, or neonatal primary outcomes were seen.” It is not specified in the text what neonatal primary outcomes are, the primary outcome is defined as rate of vaginal delivery.

**Authors’s response**: This has been rephrased: “No difference in complications, such as post-partum hemorrhage, low 5-minute Apgar score, fetal asphyxia or admission to neonatal intensive care unit were seen” (lines 302-304).

**Minor issues:**

8. Results page 3 line 24 and page 14 line 193: “p=0.004” whereas in table 4 it is p<0.004.

**Authors’s response:** Thank you, this has been corrected

9. Page 11 lines 145-146 “Neonatal infections were by a neonatologist categorized...”. This could be rephrased to “Neonatal infections were categorized by a neonatologist...”.

**Authors’s response:** We have rephrased this as suggested (lines 134-135).

10. Page 17 lines 239-240: There is a word missing “A recent review by described”

**Authors’s response:** Thank you, this has been corrected as “A recent review by Dasanayakea and Goonewardene described..” (lines 233-234).

11. Figure 2: please add labels to axes

**Authors’s response:** The axes of Fig 2 have now been labeled

12. Figure 3: label years axis. I would also suggest adding a dotted line under the arrow to show more precisely when the new oxytocin protocol was implemented.

**Authors’s response:** We have added label to the x-axis and a dotted line under the arrow in Fig 3.

**Reviewer #2:**

**Minor revisions:**

1. I note that there were several changes made to the institutional protocol, in addition to the dose of oxytocin, which could potentially contribute to the differences between groups outlined in the results section. These included 1) removal of oxytocin breaks from the protocol 2) instituting a practice of stopping oxytocin once active labour is achieved and 3) instituting a maximum duration of induction before it was considered to have failed. It is possible that these policy changes could have also contributed to the improved outcomes the authors demonstrate in the new protocol, in addition to the differences in oxytocin dosing.
While these protocol changes are outlined by the authors in the Discussion section, it should be acknowledged that these may be confounding the study findings. The conclusion currently reads that the improved outcomes seen with their protocol change were the result of higher dose alone. I think that the Discussion could be modified slightly to highlight the possible contribution of these confounding factors and that the conclusion should reflect the change in protocol which includes but is not limited to a change from low dose to higher dose oxytocin. I don't think this takes away from the overall important findings of the paper.

**Authors’s response:** Thank you for this excellent comment. We have now highlighted these confounding factors as a part of the new protocol in the Discussion and in the Conclusion (Discussion lines 226-229 and 261-264, Conclusion lines 298-300 and 307-309). We also edited the conclusion paragraph of the Abstract accordingly (lines 24-28).

2. The other point that might bear acknowledging in the manuscript is that "low dose" and "high dose" are relative terms in these types of studies. The "high dose" used here is lower than that used in other published studies. Lack of standardized dosing is one of the difficulties with the literature in this area, including the Cochrane review and other systematic reviews and meta analyses previously published. The findings here are specific to an institutional change from one protocol to another, which is important, particularly as it showed a clear benefit in terms the primary and secondary outcomes, but this finding may not be generalizable. I appreciate that the authors shared their protocol in its entirety, which would make adaptation by other institutions feasible.

**Authors’s response:** Thank you for pointing this out. The authors acknowledge our “high” oxytocin dose still remains lower compared to several other institutions, the Cochrane review and the recent European guideline. However, at the time of implementation of the new protocol in Finland, our current dose was considered extremely high compared to other national institutions. We have now discussed this topic and the lack of standardized dosing in the Discussion (lines 288-295). Encouraged by the results of this study, we are considering updating the maximum oxytocin dose of our institutional protocol higher, similar to the recommendation in the current European guideline (lines 293-295).

We thank the reviewers for these valuable comments which have much improved the manuscript.

Yours sincerely,

Heidi Kruit (Corresponding author)

Department of Obstetrics and Gynecology, Helsinki University Central Hospital

Email:heidi.kruit@hus.fi

Telephone: +358407317068
